# Cloning and prokaryotic expression of *WRKY48* from *Caragana intermedia*

**DOI:** 10.1515/biol-2022-0016

**Published:** 2022-03-07

**Authors:** Jinhua Liu, Ruigang Wang, Guojing Li, Yongqing Wan

**Affiliations:** Inner Mongolia Key Laboratory of Plant Stress Physiology and Molecular Biology, Inner Mongolia Agricultural University, Hohhot, 010018, P. R. China; Inner Mongolia Enterprise Key Laboratory of Tree Breeding, Mengshu Ecological Construction Group Co., Ltd., Hohhot, 011517, P. R. China; Inner Mongolia Engineering Research Center for Plant Gene Resources Mining and Molecular Breeding, Inner Mongolia Agricultural University, Hohhot, 010021, P. R. China

**Keywords:** bioinformatics analysis, *Caragana intermedia*, CiWRKY48, prokaryotic expression system

## Abstract

*Caragana intermedia* (*C. intermedia*) is a kind of drought-tolerant leguminous shrub. WRKY transcription factors are one of the largest family of transcription factors in plants and play critical regulatory roles in stress tolerance and the development of plants. In our study, *CiWRKY48* was cloned from *C. intermedia*, analyzed using bioinformatics software, and expressed with a prokaryotic expression system. The results showed that the open reading frame (ORF) of *CiWRKY48* was 1158bp, the molecular weight (MW) was 42 kDa, and its subcellular localization was in the nucleus. Additionally, fusion protein was obtained, and confirmed by western blotting. The stress resistance of the pET30a-His-MBP-CiWRKY48 transformed *Escherichia coli* expression strain was reduced under mannitol and salt treatment, compared with the control. Overall, our findings provided a foundation for uncovering the function of CiWRKY48.

## 1 Introduction

WRKY transcription factors are among the largest family of transcription factors in plants, and are named because of the highly conserved WRKY domain, containing 60 amino acids. According to the number of WRKY domains at the amino terminus and the types of zinc lipid domains at the carboxyl terminus, they were classified into three categories. The first category had two WRKY and C2H2 zinc lipid domains, the second category had one WRKY and C2H2 zinc lipid domain, and the third category had one WRKY and C2HC zinc lipid domain. The second type can be further subdivided into five subclasses from IIA to IIE based on other conserved amino acid sequences except for the WRKY domain [1]. The primary function of WRKY is to regulate the expression of downstream genes, so as to regulate the response of plants to the external environment [2]. It then plays an important regulatory role in plant response to various abiotic stresses [3,4], disease resistance [5], metabolism [6], and development[7,8].

The *Escherichia coli* (*E. coli*) expression system (prokaryotic expression system) is universal because it allows for high levels of heterologous protein expression [9], has a high growth rate on inexpensive substrates, and is relatively simple to operate [10]. Due to the above advantages, many heterologous proteins [11,12,13] are expressed in this system to study their functions. However, there are also some problems with the system, such as protein does not readily form in the supernatant and ubiquitously form inclusion bodies. In order to improve protein expression, in addition to changing the induction conditions, such as lowering the induction temperature and inducer concentration, some fusion tags can also be added to the prokaryotic expression vector or protein sequence, such as GST, NusA, MBP, and SUM [14]. As a result, the protein expressed in the host by these tags usually will be highly soluble. In addition, fusion tags are used to detect and purify the target protein and sometimes help transport the target protein to the periplasm to improve the biological activity of the target protein. Thus, we chose pET30a-His and pET30a-His-MBP ligated with fusion tags of MBP ligated with CiWRKY48 to form fusion protein, respectively, to ensure successful protein expression.

*C. intermedia*, commonly known as a bush forage, belongs to the legume family, grows in arid and semi-arid regions, and has resistance to adversity with high economic and ecological value [15]. WRKY transcription factors have a variety of biological functions, and currently, 53 WRKY transcription factors have been found in the transcriptome database of *C. intermedia*. Analysis of the expression pattern of *CiWRKY48* indicates that it may be involved in response to abiotic stress in previous studies [16]. Therefore, it is necessary to study CiWRKY48. Nowadays, plant expression systems will take a long time to express proteins, so the prokaryotic expression system was selected to express CiWRKY48, allowing us to understand its properties and functions. In our study, we primarily analyzed the properties of CiWRKY48 using bioinformatics software and constructed the fusion expression vector of pET30a-His-CiWRKY48 and pET30a-His-MBP-CiWRKY48 to express the protein in the prokaryotic expression system to lay the groundwork for the research of CiWRKY48.

## 2 Materials and methods

### 2.1 Bacterial strains, plasmids, and reagents

*Trans*1-T1 phage-resistant chemically competent cells of *Escherichia coli* (*E. coli*) colony strain and *pEASY* ^®^-Blunt Simple Cloning vector were purchased from TransGen Biotech. C^+^ chemically competent cells of *E. coli* expression strain, pET30-His-MBP, and pET30-His-MBP expression vector were from in our laboratory. The RNAprep pure Plant kit (DP432), the Plant Genomic DNA kit (DP305), and the common agarose gel DNA recovery kit (DP209) were purchased from TIANGEN. PrimerSTAR Max DNA polymerase and T4 DNA polymerase were purchased from TaKaRa.

### 2.2 Culture conditions of plants

The material of this study was *C. intermedia* cultured in the greenhouse. The culture conditions were as follows: vegetative soil and vermiculite (V/V = 1:3), a long-day photoperiod (16-h light/8-h dark), relative humidity of about 30%, and culture temperature of about 25°C. When the seedlings of *C. intermedia* grew for 3 weeks, the aboveground part of each plant was taken and frozen with liquid nitrogen and stored at −80°C.

### 2.3 Gene cloning and vector construction

DNA and RNA were extracted from *C. intermedia,* and RNA was reversely transcribed into cDNA. The target gene fragment was amplified by polymerase chain reaction (PCR) using DNA and cDNA as templates. Primer Sequence, forward primer 5′-TACTTCCAATCCAATGCCATGGAGGAGAAGAGAGAG-3′, and reverse primer 5′-TTATCCACTTCCAATGTTATGATCTTCCTTCTCCAGC-3′ were synthesized by Sangon Biotech. PCR products were purified and then linked to a cloning vector transformed into *Trans*1-T1 cells for sequencing. The empty expression vector was digested and linearized by *Ssp*I. Both the linear vector and the PCR product were then treated with T4 DNA polymerase and the two treated fragments were mixed and linked by natural annealing.

### 2.4 Bioinformatics analysis

The amino acid sequences were obtained from NCBI, including *Arabidopsis thaliana* WRKY48 (AtWRKY48), *Gossypium hirsutum* WRKY48 (GhWRKY48), *Populus tomentosa* WRKY48 (PtWRKY48), *Spatholobus suberectus* WRKY48 (SsWRKY48), and *Zea mays* WRKY48 (ZmWRKY48). The *Caragana intermedia* WRKY48 (CiWRKY48) nucleotide sequence was obtained from the transcriptome database in our Laboratory Then, it was translated into amino acid sequences by the software of primer premier 5. Other software programs required in this study were DNAMAN, MEGA7, ProtParam, ProtScale, NetPhos 3.1 Server, WoLF PSORT, WISS-MODEL, and Novo-Pro.

### 2.5 Protein prokaryotic expression

First, the fusion expression vectors pET30a-His-CiWRKY48, pET30a-His-MBP-CiWRKY48, and pET30a-His-MBP were transformed into *Trans*1-T1 cells for amplification and then transformed into C^+^ cells for expression of the protein. The bacteria were cultured in LB (50 µg/mL Kana) overnight (shaking table at 37°C, 200 rpm). Then, the cultivation was extended with a scale of 1:100 for 3 h to induce protein expression with 0.25 mM IPTG, followed by further culturing for 12 h (16°C, 200 rpm). After that, bacteria were collected by centrifugation and was resuspended in NTA0 buffer. The bacteria were broken by ultrasonication (4°C), and then the supernatant and pellet were collected by centrifugation (4°C); the protein was purified by Ni-NTA resin. Finally, the supernatant, pellet, and the purified product were separated for electrophoresis on 10% SDS-PAGE. These proteins were visualized using Coomassie brilliant blue (CBB) staining. Finally, the image was saved using an Amersham Imager 600 (AI600).

### 2.6 Western blotting (WB)

The purified protein (20 µL; added 5 µL of 5× loading buffer) was boiled for 5 min to perform electrophoresis on 10% SDS-PAGE. The next steps were as follows: transferring the PVDF membrane, blocking (TBST buffer containing 5% milk m/v), incubating with first antibody (His-Tag antibody, 4°C overnight), washing with TBST (6 times, 5 min each time), incubating with secondary antibody (shaking table at 25°C, 70 rpm, 2 h), washing with TBST, adding enhanced chemiluminescent (ECL) and observing the PVDF membrane using a AI600 for chemiluminescence.

### 2.7 Stress resistance test of expression strain

Expression strain (transformed with the fusion expression vectors pET30a-His-MBP-CiWRKY48 and pET30a-His-MBP) was cultured in LB (50 µg/mL Kana) to OD_600_ = 0.8, and IPTG (0.25 mM) was added for induction for 1 h. After that, 200 µL of the bacteria solution was taken to a 20 mL LB stress medium (0, 200, 300, 400, 500 mM NaCl; 0, 400, 500, 800, 1,000 mM mannitol) for culturing for 12 h. The OD_600_ value of the bacteria solution was measured with a spectrophotometer.

## 3 Results

### 3.1 *CiWRKY48* cloning and sequence analysis

**Figure 1:**
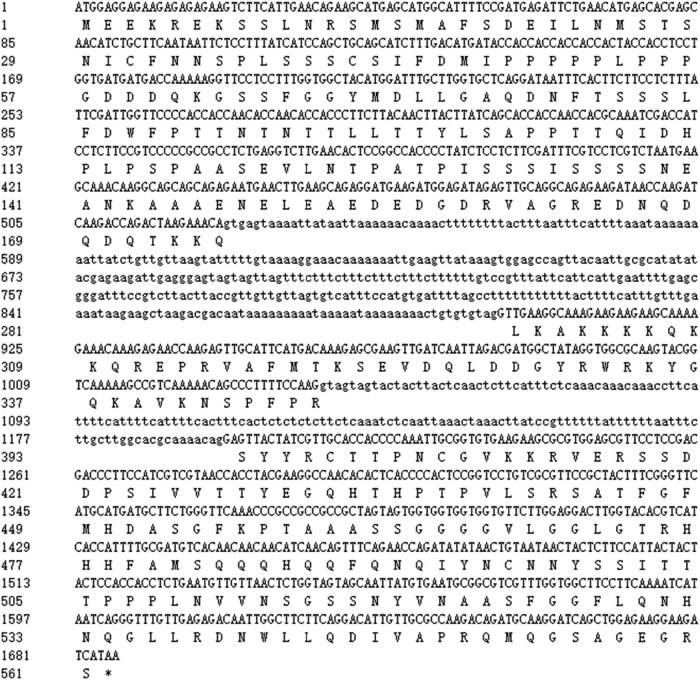
Nucleotide sequence and amino acid sequences of CiWRKY48: the upper row of line numbers represents the sequence of nucleic acids (the capital letters represent the sequence of exons and the lowercase letters represent the sequence of introns) and the lower row represents the sequence of amino acids.

A high-fidelity enzyme PrimeSTAR was used to amplify *CiWRKY48*, and PCR products were purified using a common agarose gel DNA recovery kit to attach the cloning vector for sequencing by Sangon Biotec. The sequencing results were analyzed with software (DNAMAN), and showed that the full length gDNA of *CiWRKY48* was 1,686 bp, containing 2 introns, and ORF was 1,158 bp, encoding a protein of 385 amino acids as shown in Figure 1.

### 3.2 Bioinformatics analysis

**Figure 2:**
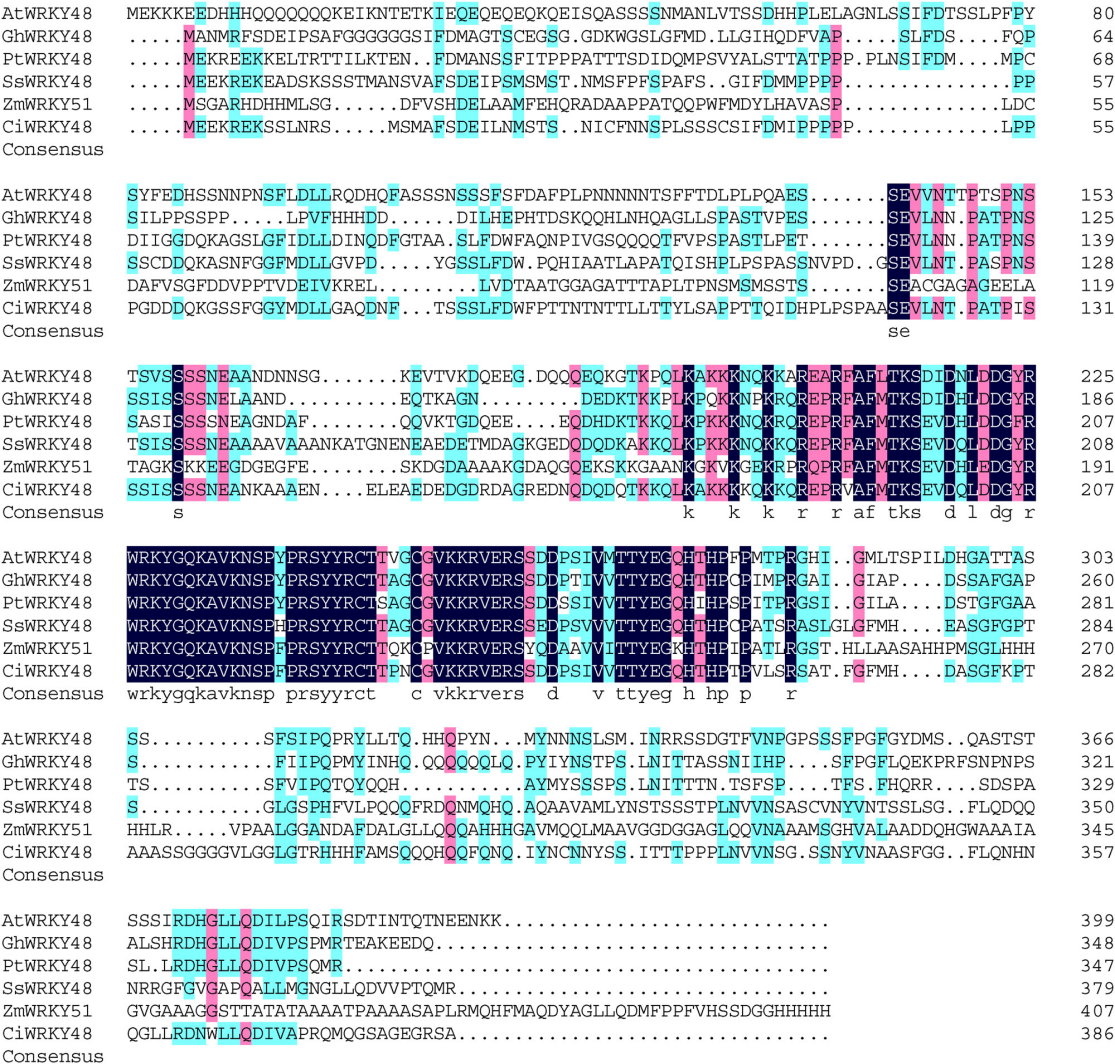
Multiple sequence alignment of CiWRKY48 with its homologs from other species.

**Figure 3:**
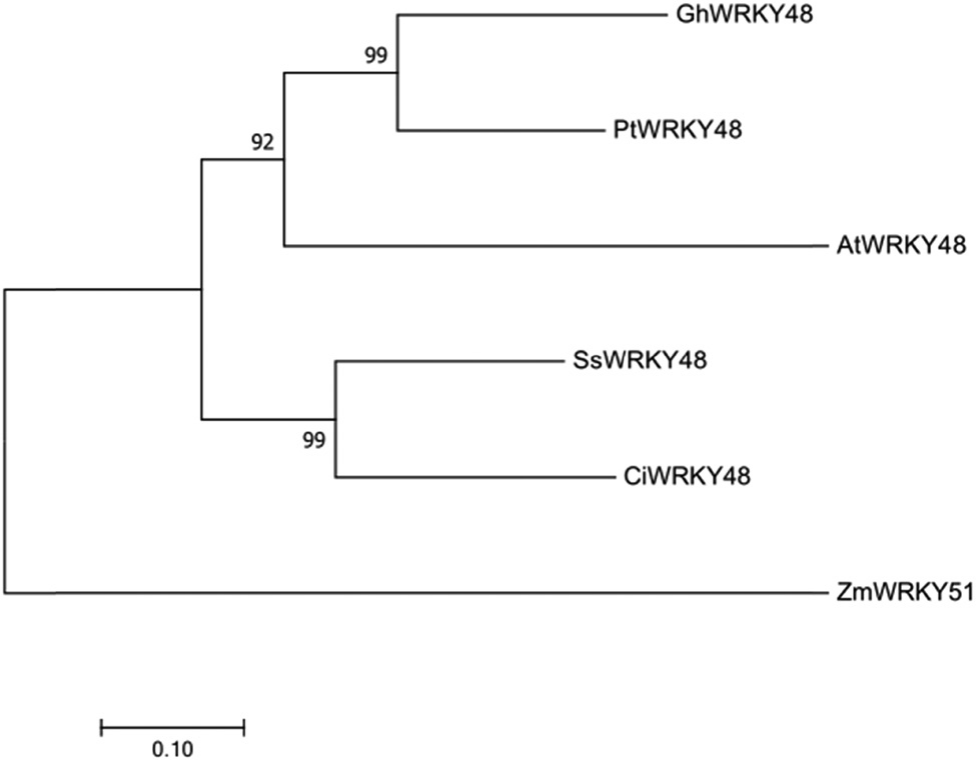
Phylogenetic analysis of CiWRKY48 with its homologs from other species.

Multiple sequence alignment with DNAMAN showed that the amino acid sequences of AtWRKY48, GhWRKY48, PtWRKY48, SsWRKY48, and ZmWRKY48 had high similarity with CiWRKY48 and contained one WRKY domain and C2H2 zinc lipid domain. From previous research of phylogenetic analysis [16], it belonged to the group IIc WRKY family as shown in Figure 2. Phylogenetic analysis by MEGA7 showed that CiWRKY48 had the highest similarity to SsWRKY48 as shown in Figure 3, and CiWRKY48 and SsWRKY48 were all part of the legume WRKY proteins.

**Figure 4:**
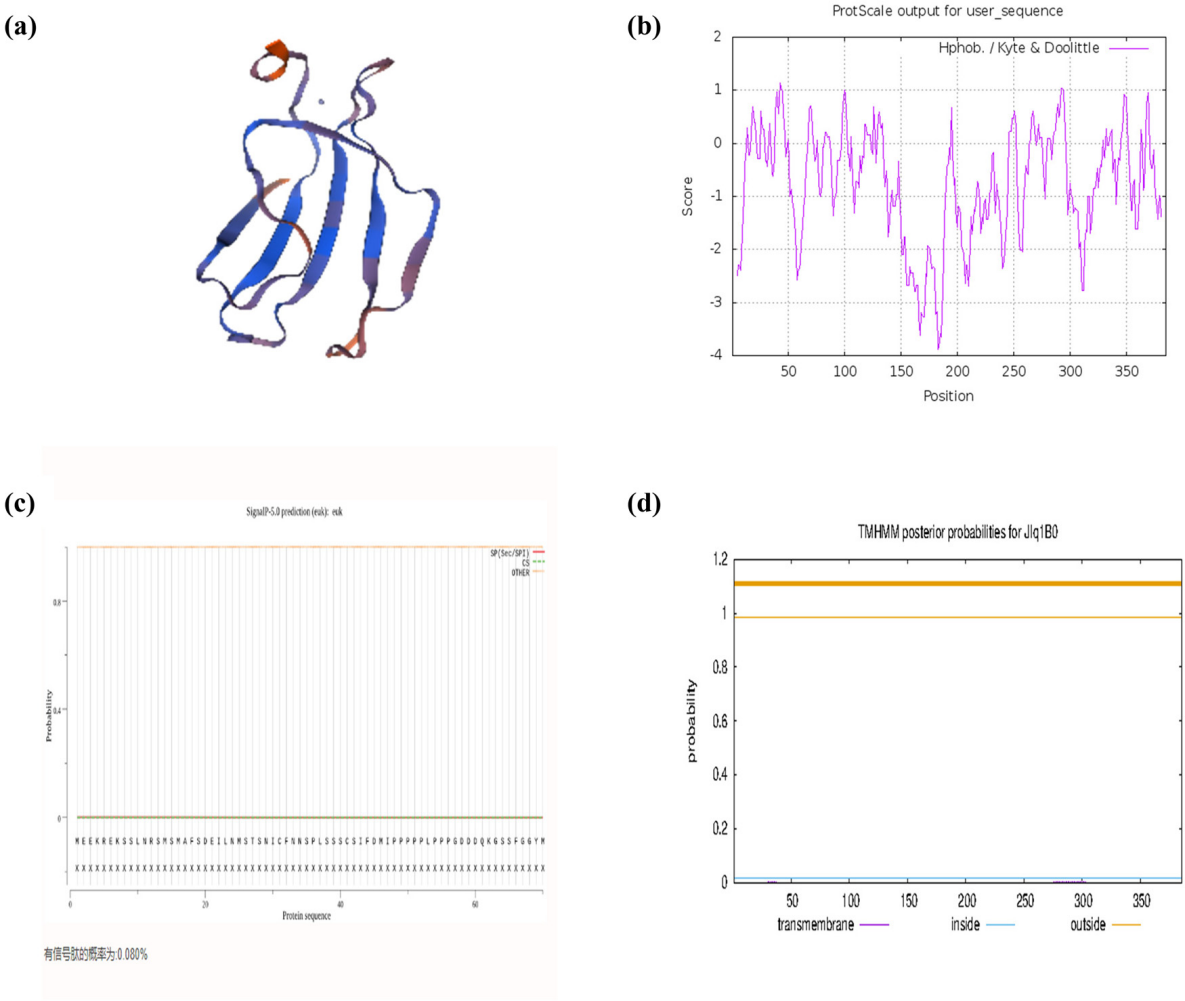
Bioinformatics analysis of CiWRKY48: (a) The 3D structure prediction diagram; (b) phosphorylation site prediction map; (c) signal peptide prediction map; and (d) transmembrane doamain prediction of CiWRKY48.

The physical and chemical properties of the CiWRKY48 protein were predicted by ProtParam, ProtScale, and NetPhos 3.1 Server. The results show that the molecular weight (MW) of CiWRKY48 was 42 kDa, the protein instability index was 59.07 U, the theoretical pI was 6.15, and the hydrophilic average coefficient was −0.806, and, according to the rule that the lower the amino acid score is, the stronger the hydrophilic and the higher the score is, the stronger the hydrophobicity is, the amino acids between +0.5 and −0.5 are mainly amphoteric amino acids, so the protein is a hydrophilic protein. The amino acid phosphorylation sites of WRKY48 contained 35 serines, 14 threonines, and 4 tyrosines, as shown in Figure 4.

The subcellular localization by WoLF PSORT indicated that WRKY48 was localized in the nucleus. The three dimensional (3D) structure prediction of WRKY48 protein by the WISS-MODEL showed that the protein was made up of five β-sheet (Figure 4). In addition, the present rate of the predicted signal peptide sequence was very low (only 0.08%), and there was no transmembrane domain by Novo-Pro prediction (Figure 4).

### 3.3 Prokaryotic protein expression and WB analysis

**Figure 5:**
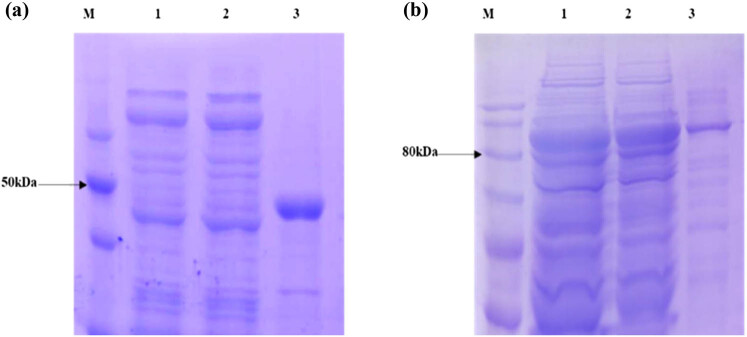
The SDS-PAGE gel electrophoresis of the expressed pET30a-His-CiWRKY48 and pET30a-His-MBP-CiWRKY48 proteins (a) pET30a-His-CiWRKY48; (b) pET30a-His-MBP-CiWRKY48 protein expression, M, Maker; lanes 1-2, supernatant; and lane 3, pellet.

In our study, we chose pET30a-His and pET30a-His-MBP vectors with CiWRKY48 to form recombinant proteins, respectively, and found that the former is expressed only in the precipitate, but the latter was expressed in both supernatant and pellet. In total, the latter had a better-expressed effect, as shown in Figure 5.

**Figure 6:**
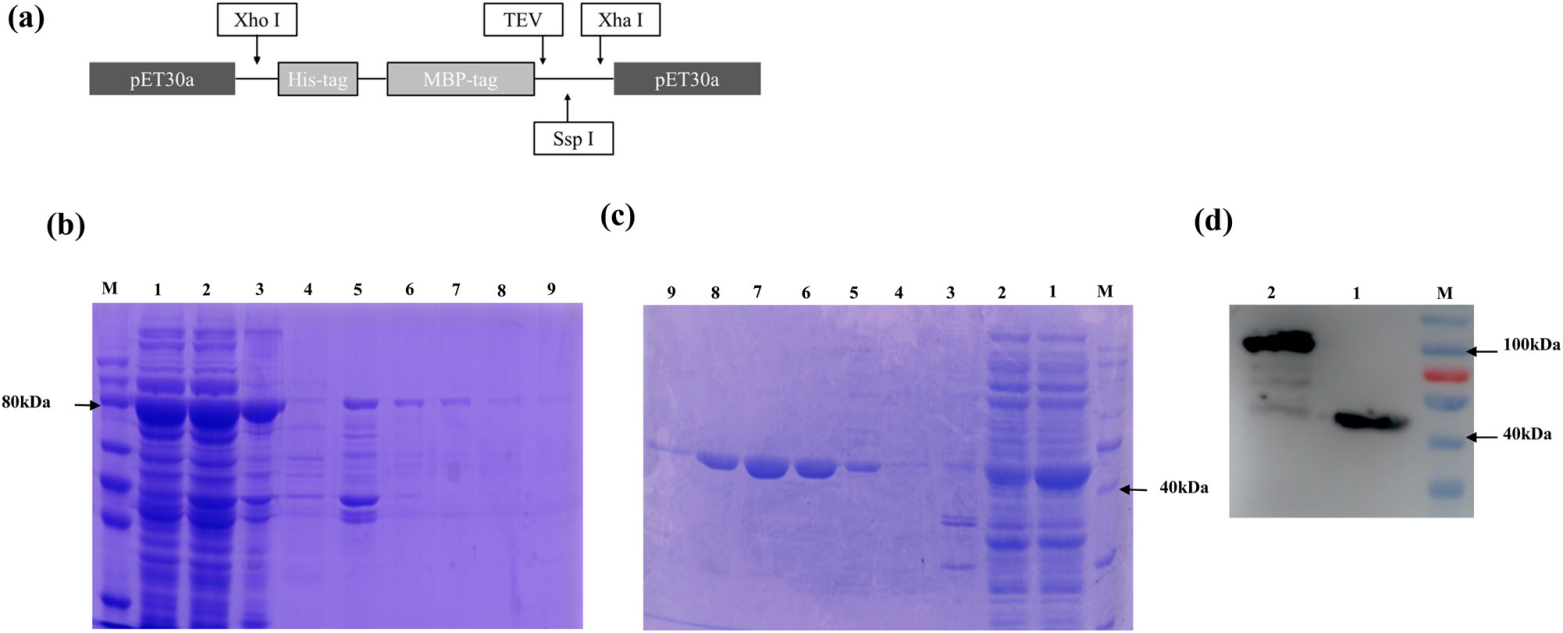
Purification of His-MBP-CiWRKY48 and His-MBP protein as well as the WB verification. (a) The position of each element on the plasmid pET30a; (b) the SDS-PAGE of the purified pET30a-His-MBP-CiWRKY48 protein; (c) the SDS-PAGE of the purified pET30a-His-MBP protein, M, Maker; lane 1, supernatant; lane 2, the flow through; lane 3, pellet; lane 4, NTA0; lane 5, NTA10; lane 6, NTA30; lane 7, NTA50; lane 8, NTA100; lane 9, NTA200; (d) the WB of the purified His-MBP-CiWRKY48 and His-MBP proteins. M, Maker; lane 1, His-MBP protein; lane 2 NTA50-eluted His-MBP-CiWRKY48 protein.

These two fusion proteins, pET30a-His-MBP-CiWRKY48, and pET30a-His-MBP (used as the control), containing a histidine tag, respectively, were successfully induced and expressed in the prokaryotic expression system. They were purified through the Ni-NTA resin and verified by WB with His-Tag antibody. The results showed that the fusion target proteins pET30a-His-MBP-CiWRKY48 (86 kDa) and pET30a-His-MBP (44 kDa) were obtained (Fig. 6).

### 3.4 Stress resistance test of the expression strain

**Figure 7:**
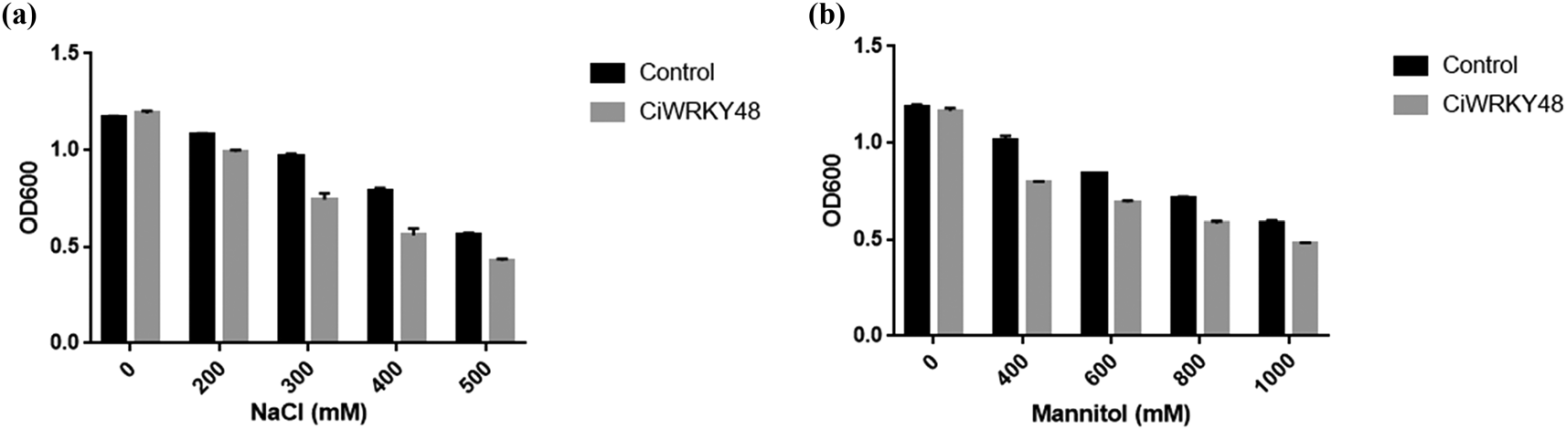
The growth status of the expression strains under salt and mannitol treatments. (a) Indicates treatment under different concentrations of NaCl ; (b) indicates treatment under different concentrations of mannitol.

The expression strain was tested for resistance to salt and mannitol treatment, as shown in Figure 7; the results showed that the *E. coli* expression strain (transformed with pET30a-His-MBP-CiWRKY48) was more sensitive than the control (transformed with pET30a-His-MBP).

## 4 Discussion

*C. intermedia* is considered an ecological restoration species due to its drought tolerance, which indicates that it plays an important role in adapting stress resistance. WRKY transcription factors are one of the largest family of transcription factors in plants and play important roles in various aspects of plants. According to the current research on the expression patterns of WRKY transcription factors in *C. intermedia,* WRKY transcription factors may play a role in stress signaling pathways [16]. Furthermore, studies about WRKY prokaryotic proteins of *C. intermedia* have not been reported. For the above reasons, the goal of this study was to understand the properties of the protein and get a preliminary understanding of its resistance.

The prokaryotic expression system is one system that was operated easily and took less time to express heterologous proteins, including plant proteins [17,18,19]. At present, a wide range of plant proteins have been expressed using this system and are directly used for functional studies [17,20] or interaction experiments *in vitro* [21,22,23]. Based on the above reasons, the CiWRKY48 protein was expressed in a prokaryotic expression system. However, to successfully express CiWRKY48, two expression vectors with different tags were linked to CiWRKY48 and transformed into the protein-expressing strain. The results demonstrated that the protein with fusion tags of Mbp could be expressed more effectively. Following that, the protein was purified using the Ni-NTA resin and verified by WB; according to the WB verification results, the target protein was indeed expressed. To better understand the target protein’s resistance, the expression strain was tested for the resistance towards mannitol and salt, and the result revealed that the expression strain was sensitive to mannitol and salt. CiWRKY48, as a plant protein, may have its own special stress response mechanism in a plant, so it is necessary to conduct further functional studies in a plant expression system. In conclusion, CiWRKY48 was mainly expressed in the prokaryotic expression system in our study, which will help us understand the basic properties of the protein and lay a foundation for functional studies or interaction experiments *in vitro*.

## 5 Conclusion

The protein CiWRKY48 was successfully induced and expressed with a prokaryotic expression system, and conducted a stress resistance test of the expression strain. These above results laid a foundation for the functional study of this protein.
